# Oxidative stability of lipid fractions of sponge-fat cakes after green tea extracts application

**DOI:** 10.1007/s13197-019-03750-5

**Published:** 2019-04-13

**Authors:** Mariola Kozłowska, Anna Żbikowska, Arkadiusz Szpicer, Andrzej Półtorak

**Affiliations:** 1 Department of Chemistry, Faculty of Food Sciences, Warsaw University of Life Sciences (WULS-SGGW), Nowoursynowska 159C St., 02-776 Warsaw, Poland; 2 Department of Food Technology, Faculty of Food Sciences, Warsaw University of Life Sciences (WULS-SGGW), Nowoursynowska 159C St., 02-776 Warsaw, Poland; 3 Division of Engineering in Nutrition, Faculty of Human Nutrition and Consumer Sciences, Warsaw University of Life Sciences (WULS-SGGW), Nowoursynowska 159C St., 02-776 Warsaw, Poland

**Keywords:** Sponge-fat cakes, Green tea extract, Oxidative stability, Rancimat, Differential scanning calorimetry

## Abstract

Oxidative stability of lipid fractions extracted from sponge-fat cakes enriched with green tea extracts and synthetic antioxidant (BHA) directly after baking and after 28 days of storage was investigated. This was achieved by the determination of peroxide (PV), *p*-anisidine (*p*-AnV) and acid values (AV), and using Rancimat test or differential scanning calorimetry method, respectively. The results showed that the lipid fractions extracted from sponge-fat cakes containing the addition of BHA (0.02%) and green tea extract at concentrations of 1% exhibited a greater resistance to oxidation than those from cakes without additives. AV values were the lowest for lipids extracted from sponge-fat cakes enriched with 1% green tea extract up to the end of storage. The incorporation of BHA and green tea extract (1%) into cakes caused a gradual increase of PV and *p*-AnV values during 21 days of sample storage. The values of these parameters increased significantly for samples without any additives, especially in regard to PV. What is more, thermal analysis showed that samples enriched with 1% green tea extract and with BHA were characterized by higher onset temperature (t_ON_), activation energy, and induction time (τ) than samples without any additives, especially during 21 days of storage. The increase of green tea extract concentration to 1% in cookies reduced L^*^ (from 63.85 to 51.15) and b^*^ (from 34.64 to 29.11) values, while a^*^ value showed an increase from 8.43 to 11.17.

## Introduction

Green tea is one of the most popular beverages consumed around the world, especially in Asian countries (El-Anany 2013). It is prepared from leaves not subjected to fermentation of the genus *Camellia* as an infusion with a pleasant taste that can be improved by the addition of different fruits and spices. Leaves of green tea contain bioactive molecules that bring a variety of health benefits. There are polyphenols, flavonoids such as flavanols (catechins, procyanidins), flavonos (rutin, quercetin, kaempferol) and phenolic acids (gallic, caffeic) (Gramza-Michałowska et al. 2016; Lorenzo and Munekata 2016). Most of the total polyphenol content is constituted by catechins (85%), mainly: (+)-catechin (C), (−)-epicatechin (EC), (−)-gallocatechin (GC), (−)-epicatechin gallate (ECG), (−)-epigallocatechin (EGC) and (−)-epigallocatechin gallate (EGCG) (Oliveira 2012; Nikoo et al. 2018). Green tea polyphenolic catechins inhibit growth of a wide spectrum of Gram-positive and Gram-negative bacteria species with moderate potency (Taylor et al. 2005) and also demonstrate antioxidant activity (Gramza et al. 2005a; Saito et al. 2009). Application of green tea phenolic compounds to foods might prevent the formation of free radicals and inhibit lipid oxidation and microbial spoilage, which affect the quality of products and decrease their shelf life (Namal Senanayake 2013; Thomas and Wansapala 2017; Mir et al. 2018). Changes in foods caused by lipid oxidation also include loss of colour and of nutrient value and accumulation of compounds which may be detrimental to consumers’ health (Wąsowicz et al. 2004). Particularly susceptible to oxidative changes are fats with a high content of unsaturated fatty acids, especially when subjected to thermal treatment. Fat also plays an important role of a raw material and a functional ingredient for many food products such as bakery, confectionery, shortenings, and margarines. It may modify sensory characteristics of a product by changing the physical properties of the dough and ultimately the texture of the finished product, improve the porosity of the crumb and the stability of the dough during processing in forming machines, during its growth and at the initial baking stage (Katyal et al. 2019; Mamat and Hill 2014; Rios et al. 2014). In the formulation of sponge-fat cakes, 25–40% fat is added to improve taste, crispness and friability of finished products and to delay the staling process. Large amounts of fat in these kinds of food products may promote oxidation, hydrolysis of glycerides, and formation of tr*ans* isomers of fatty acids (Miśkiewicz et al. 2013). Since lipid oxidation is an important factor determining the quality of food products, including sponge-fat cakes, many studies are carried out to inhibit this process by using antioxidants, especially of natural origin, such as plant extracts (Mišan et al. 2011; Reddy et al. 2005). They are preferred by consumers more than synthetic antioxidants due to their health benefits and the possibility of enriching food products with ingredients that improve their sensory and physicochemical properties. The objective of the present study was to determine oxidative stability of lipid fractions extracted from sponge-fat cakes enriched with green tea extracts or BHA directly after baking and after storage of cakes in thermostated conditions at 63 °C for 28 days.

## Materials and methods

### Materials

Aqueous green tea extract in the form of a lyophylisate, which was used as a fortification agent, was provided by Goldmann (Warsaw, Poland). The other cake dough ingredients were: emulsified fat—“Rama Culinese” margarine containing 82% fat (Unilever Poland), wheat flour type 480, potato flour (Kupiec, Poland), sugar powder, eggs, and baking powder (local market).

### Reagents

Potassium hydroxide, potassium iodide, sodium thiosulfate, starch soluble, phenolphthalein, ethanol, methanol, diethyl ether, chloroform, acetic acid (glacial), *n*-hexane were purchased from POCH (Gliwice, Poland). Butylated hydroxyanisole (BHA) and 2,2-diphenyl-1-picrylhydrazyl (DPPH) were obtained from Sigma-Aldrich Chemicals (Poznań, Poland). All solvents and reagents were analytical grade and were used without additional purification.

### Antioxidant activity of the green tea extract and BHA

The antioxidant activity of the samples was measured using the free radical, 2,2-diphenyl-1-picrylhydrazyl (DPPH) according to the method described by Gow-Chin and Hui-Yin (1995) with some modifications. To the 0.2 mL of previously prepared 0.5 mg/mL antioxidant stock solutions (water green tea extract solution and ethanol BHA solution, respectively) 1 mL of freshly prepared DPPH-methanol solution (0.3 mM) was added. Then, 3.8 mL of methanol was added to each sample tube and the mixture was shaken vigorously. After 10 min of samples’ incubation in the dark at room temperature, the absorbance at 517 nm was measured. The control was prepared, as above, without any antioxidant and methanol was used for the base line correction. The percentage of DPPH inhibition was calculated according to the formula: DPPH inhibition (%) = [(Abs control − Abs sample)/Abs control] × 100%.

### Preparation of the sponge-fat cakes

Powdered sugar (90 g) and fat (95 g) were creamed for 6 min using Braun K650 Multiquick kitchen machine food processor (Germany). Eggs (140 g) were added to this smooth cream, and the mixture was blended for a further 2 min. Then wheat flour (110 g) sieved twice and baking powder (10 g) were added and mixed for 7 min to obtain a homogeneous dough. The dough (350 g) was transferred to aluminium trays and placed in the Unox convection oven (model XBC, Vigodarzere, Italy) and baked at 160 °C for 45 min. The cake samples were also prepared in four formulations, i.e. with the addition of a synthetic antioxidant (BHA 0.02%) and 3 different concentrations of green tea extracts: 0.02%, 0.2%, and 1% (relative to the fat mixture). The baked dough was cooled to room temperature, packed in plastic pouch (PEHD) and thermostated (ELKON CWE-4a thermostat, Łódź, Poland) to conduct an accelerated storage test in a force-draft oven at 63 °C for 28 days. Samples were withdrawn after 0, 7, 14, 21 and 28 days of storage.

### Colour characteristics

The colour intensity of sponge-fat cakes with and without the addition of different concentrations of green tea extracts and BHA was determined after baking using a tristimulus reflectance colorimeter (Minolta CM-3600d, Konica Minolta Sensing, Inc., Tokyo, Japan). It was expressed as L*, a* and b*, where L* represents whiteness of colour (value 100) or blackness (value 0), a* represents red (positive value) or green (negative value), and b* defines the proportion of yellow (positive value) or blue (negative value). The final result was the arithmetic mean of 12 measurements. Colour difference between samples containing antioxidants and the control sample was also calculated as (ΔE) = [(L_c_^* ^− L_x_^*^)^2^ + (a_c_^* ^− a_x_^*^)^2^ + (b_c_^* ^− b_x_^*^)^2^]^1/2^, where L_c_^*^, a_c_^*^, b_c_^*^ are the colour parameters of the control sample and L_x_^*^, a_x_^*^, b_x_^*^ are the colour parameters of the samples containing antioxidants.

### Lipid extraction

The lipid fractions were extracted from the sponge-fat cakes according to the Folch et al. method (1957) using a mixture of solvents such as chloroform and methanol (2:1; v/v) in a laboratory shaker under ambient conditions. After filtration and separation of lipid fractions, the solvents were removed by evaporation under reduced pressure using rotary evaporator (Rotavapor R-215, Büchi Labortechnik, Switzerland) at 40 °C. Chemical analysis of the cakes’ lipid fractions consisted of determination of acid value (AV), peroxide value (PV), *p*-anisidine value (*p*-AV), Totox value, and of Rancimat and differential scanning calorimetry (DSC) measurements.

### Chemical analysis

The acid, peroxide, and *p*-anisidine values were determined according to ISO standard methods (660:^2009^, 3960:^2009^, 6885:^2008^, respectively). Acid values were determined by titration of lipid fractions samples dissolved in a mixture of ethanol:diethyl ether (1:1; v/v) with 0.1 M ethanolic potassium hydroxide solution using phenolphthalein indicator to the pink colour persisiting for at least 10 s. The results of AV were expressed as mg KOH per gram of fat sample (mg KOH/g) and were calculated according to the equation: AV = (V × 5.611)/m, where V is the volume (mL) of sodium hydroxide titrant used and m is the mass of lipid fraction sample (g). Peroxide values were determined by titration of lipid fractions samples dissolved in a mixture of chloroform:glacial acetic acid (2:3; v/v) in the presence of saturated potassium iodide solution and starch as an indicator with 0.02 M sodium thiosulphate solution from a purple to a slight yellow or colourless endpoint. The results of PV were shown in miliequivalent of active oxygen per kg of fat sample (meq O_2_/kg) and were calculated according to the equation: PV = (V− V_0_) × c/m where V and V_0_ are the volume (mL) of sodium thiosulphate exhausted by test sample and blank, respectively, m is the mass of lipid fraction sample (g), and c is the concentration of sodium thiosulphate (mM). The *p*-anisidine values were measured spectrophotometrically in quartz cuvettes with the 10 mm optical path length on a Helios Gamma UV–Vis Spectrophotometer (USA). The lipid fractions samples were dissolved in isooctane and 5 mL of this solution was transferred to two test tubes. To one of them *p*-anisidine reagent and to the second glacial acetic acid were added, respectively. The third tube contained 5 mL of isooctane and *p*-anisidine (blank). After 8 min, the absorbance of all three samples was measured at 350 nm against isooctane. *p*-AV was calculated according to the equation: *p*-AV = (100 × Q × V/m) × [1.2 × (A_1 _− A_2 _− A_0_)] where V is the volume (mL) of the solvent in which the test sample was dissolved, Q is the content of the sample in the measured solution from which the *p*-anisidine number is expressed, m is the mass of lipid fraction sample (g), A_1_ is the absorbance of the fat solution after reaction with the *p*-anisidine reagent, A_2_ is the blank absorbance and A_0_ is the absorbance of the fat solution. Totox index was calculated on the basis of peroxide and *p*-anisidine values (Totox = 2 PV + *p*-AV).

### Thermal stability of lipid fractions

Thermal stability of the lipid fractions was evaluated by means of DSC and Rancimat measurements using Mettler Toledo DSC apparatus model 820 (Schwerzenbach, Switzerland) with air flow of 60 ml min^−1^ and Methrom Rancimat apparatus model 743 (Herisau, Switzerland) under a constant air flow (20 L/h), respectively. For purposes of the Rancimat measurements, fat samples extracted from the sponge-fat cakes after baking and storage for 28 days were weighed (2.5 g) into a reaction vessel and heated to 120 °C. The induction time for oxidation was expressed in hours (h). Meanwhile, the stability of fat samples extracted from the sponge-fat cakes after baking and storage for 7, 14, 21 and 28 days was monitored using the non-isothermal DSC technique based on heating fat samples weighing 3.6–4.0 mg in aluminium pans at the heating rates of 4, 7.5, 10, 12.5, 15 °C min^−1^, respectively. Each fat sample tested and the reference sample (pan left empty) were placed into an aluminium pan and covered with a lid with a hole drilled in its center in order to allow the samples to be in contact with the air stream; they were placed then into the DSC cell. They were heated linearly to 300 °C. Onset oxidation temperatures (t_ON, °C_) were determined as the intersection of the extrapolated baseline and the tangent line of the resulting oxidation exotherms. t_ON_ experimental values, as a function of heating rates (*β*), were directly measured and recalculated as absolute onset temperatures (T_ON_, K). Using the Ozawa–Flynn–Wall method and the Arrhenius equation, the kinetic parameters of the oxidation process (activation energy Ea, pre-exponential factor Z, and induction time τ) were calculated. The calculation procedures for the kinetic treatment were given in a recent study (Kozlowska et al. 2013; Kozłowska and Gruczyńska 2018).

### Statistical analysis

A one-way analysis of variance (ANOVA) and Tukey’s test were used to establish the significance of differences between the means at *p *< 0.05. Statistical analysis was carried out with Statgraphics plus 4.0 package (Statistical Graphics Corp., USA).

## Results and discussion

### Antioxidant activity of green tea extract and BHA

The DPPH radical scavenging assay was used for the rapid evaluation of the green tea extract and BHA antioxidant activity. The green tea extract had stronger radical scavenging activity compared with BHA. The percentage of DPPH inhibition caused by green tea extract was 83%, while the DPPH scavenging by BHA was 52%. This is largely because, green tea extract is a very good source of active antioxidant compounds like polyphenols, mainly catechins. Catechins are flavan derivatives and in a group of flavonoids they are distinguished by the highest oxidation degree of heterocyclic ring and by good solubility in water (Gramza et al. 2005a). Gramza et al. (2005b) also found that green tea extract showed higher DPPH^∙^ radicals scavenging activity than BHA and ascorbic acid. The higher than BHT and α-tocopherol abilities of DPPH^∙^ free radicals scavenging was also affirmed for catechin, quercetin and rutin. Huyut et al. (2017) showed that 3,4-dihydroxy-5-methoxybenzoic acid exhibited more DPPH radical scavenging activity than the reference antioxidants BHT, α-tocopherol and trolox but similar to that of BHA.

### Colour characteristic

The colour measurements showed significant difference (*p *< 0.05) between the sponge-fat cakes enriched with all levels of green tea extracts and the sample without any additives (control) (Table 1). L* and b* values showed that the control sample was more light and yellow than the sponge-fat cakes fortified with antioxidants. These values were significantly decreased as green tea extracts became part of the recipe for sponge-fat cakes. The sponge cakes with the addition of 0.02% of green tea extract were characterized by greater lightness (63.85) and yellowness (34.64) than cookies with higher addition of extracts. The increase in concentration of green tea extract to 1% reduced L* to 51.15 and b* to 29.11 but a* increased from 8.43 to 11.17. A similar tendency was also observed by Ahmad et al. (2015), where the effect of green tea powder on physical properties of cookies was investigated. Singh et al. (2016) also observed that L* and a* values of muffin crust and crumbs decreased when the level of black carrot fibre added to cakes was increased. In turn, L*, a* and b* values for cakes enriched with BHA was similar to that determined for the sample containing 0.02% of the green tea extract. The presence of green tea extracts resulted in a darker colour of cakes. It is known that the Maillard reaction plays an important role in colour formation. Many different polyphenol compounds present in tea, rosemary and cinnamon have been shown to be efficient α-dicarbonyl trapping agents, which are reactive intermediates that accelerate Maillard reaction due to their higher reactivity compared to glucose (Lund and Ray 2017). In regard to ΔE parameter, the highest ΔE value was found for the sponge-fat cakes with the addition of 1% green tea extract compared with sponge-fat cakes without antioxidant addition. Generally, the increase in concentration of green tea extract increased ΔE values and ranged from 3.73 (sample containing 0.02% green tea extract) to 17.27 (sample containing 1% green tea extract). In the case of samples enriched with BHA (2.61) compared with the control sample, the colour difference was not noticeable. According to Francis and Clydesdale (1975) study only when ΔE > 3 the difference in colour can be perceived by the human eye.

**Table 1:** Colour profiles of sponge-fat cakes enriched with green extracts and BHA after baking

Sample	L^*^	a^*^	b^*^	ΔE
Control	66.45 ± 2.89^e^	9.73 ± 0.88^c^	36.98 ± 1.73^e^	–
BHA	64.23 ± 2.56^d^	8.98 ± 0.72^b^	35.84 ± 1.76^d^	2.61
Green tea extract 0.02%	63.85 ± 2.32^c^	8.43 ± 0.67^a^	34.64 ± 0.99^c^	3.73
Green tea extract 0.2%	61.10 ± 1.75^b^	9.08 ± 0.62^d^	30.63 ± 1.63^b^	8.33
Green tea extract 1%	51.15 ± 1.13^a^	11.17 ± 0.89^e^	29.11 ± 1.44^a^	17.27

The values are mean ± SD. Mean values with different letters in the columns are statistically different at *p* < 0.05

### Chemical analysis

**Fig. 1:**
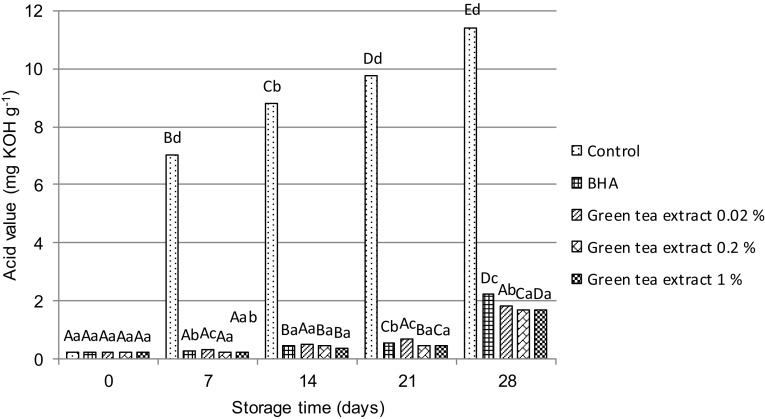
Acid value (AV) changes of the lipid fractions extracted from sponge-fat cakes enriched with different levels of green tea extracts and BHA after baking (0) and 28 days of storage. (A–E) Denotes statistically significant differences (*p* < 0.05) within group of lipid fractions extracted from the same type of sponge-fat cakes (depending on the storage time of cakes). (a–d) Denotes statistically significant differences (*p* < 0.05) within group of lipid fractions extracted from various types of sponge-fat cakes (depending on the cakes formula)

Lipid fractions extracted from the sponge-fat cakes after their baking and during storage were analysed in relation to parameters such as acid, peroxide and *p*-anisidine values, respectively. The results of these analyses are presented in Fig. 1 and Table 2. The acid value is a measure of concentration of free fatty acids appearing after triglyceride hydrolysis caused by the action of moisture, temperature, and lipolytic enzyme lipase. It is an important indicator of fat quality. In our studies, the acid value of raw fat used for baking of the sponge-fat cakes reached 0.21 mg KOH per gram of fat and it did not change significantly for lipid fractions extracted from this type of cakes directly after baking, regardless of whether they were enriched with a synthetic antioxidant—BHA or a natural antioxidant such as green tea extract (Fig. 1). However, the changes in AV values were observed for all lipid fractions obtained from the sponge-fat cakes throughout their storage for 7, 14, 21 and 28 days, respectively. AV values increased gradually up to the end of the storage time for all the samples, but the highest AV values were found for lipids extracted from the sample without the addition of antioxidants (the control sample). They changed dramatically from 0.24 (after baking) to 11.45 mg KOH/g of fat (after 28 days). In this case, the degree of hydrolysis, assessed in terms of changes in AV, may be affected by the type of fat used for preparation of sponge-fat cakes. It was an emulsified fat containing about 20% of water phase. Żbikowska and Kowalska (2007) also reported that the type of fat has a significant impact on values of this indicator. In turn, when fat was extracted from the sponge-fat cakes enriched with antioxidants, AV values were slightly higher after 14 days of storage. After 28 days, they were eight times higher compared to the control sample for the samples enriched with green tea extracts and nine times higher for those with BHA. It was determined that AV values ranged from 0.21 to 2.22 mg KOH/g of fat. The lowest tendency to hydrolyze was observed for lipid fractions extracted from the sponge-fat cakes prepared with the addition of green tea extract at concentrations of 0.2 and 1%, respectively. The natural antioxidant (green tea extract) kept the degree of hydrolysis at almost similar level as the synthetic antioxidant (Aydeniz and Yilmaz 2016).

**Table 2:** Changes in qualitative parameters (PV, *p*-AnV, Totox, Induction time) of the lipid fraction extracted from sponge-fat cakes enriched with different levels of green tea extracts and BHA after baking (0) and 28 days of storage

Storage time (days)	Control	BHA 0.02%	Green tea extract		
			0.02%	0.2%	1%
Peroxide value—PV (meq O_2_/kg)					
0	0.99 ± 0.02^Ac^	0.89 ± 0.01^Aa^	0.95 ± 0.01^Ab^	0.93 ± 0.01^Ab^	0.88 ± 0.01^Aa^
7	63.45 ± 1.34^Dc^	1.63 ± 0.02^Bb^	1.71 ± 0.02^Bb^	1.63 ± 0.02^Bb^	1.47 ± 0.02^Ba^
14	89.55 ± 2.03^Ee^	1.75 ± 0.02^Ca^	7.17 ± 0.03^Cd^	6.98 ± 0.03^Cc^	5.63 ± 0.02^Cb^
21	24.85 ± 0.05^Ce^	5.38 ± 0.03 ^Da^	22.37 ± 0.02^Dd^	12.39 ± 0.12^Dc^	11.45 ± 0.11^Db^
28	17.45 ± 0.03^Bb^	6.47 ± 0.02^Ea^	88.47 ± 2.76^Ee^	55.90 ± 1.12^Ed^	43.65 ± 1.07^Ec^
*p*-Anisidine value—*p*-AnV					
0	3.95 ± 0.02^Aa^	3.95 ± 0.08^Aa^	3.96 ± 0.01^Aa^	3.95 ± 0.02^Aa^	3.96 ± 0.01A^a^
7	14.52 ± 0.45^Bd^	4.03 ± 0.02^Ba^	4.17 ± 0.02^Bc^	4.05 ± 0.02^Bab^	4.07 ± 0.02^Bb^
14	17.13 ± 0.50^Cd^	4.08 ± 0.03^Ca^	5.47 ± 0.04^Cc^	5.47 ± 0.03^Cc^	5.11 ± 0.02^Cb^
21	37.22 ± 1.09^De^	4.54 ± 0.02 ^Da^	11.17 ± 0.05^Dd^	7.34 ± 0.02^Dc^	6.46 ± 0.03^Db^
28	66.38 ± 2.16^Ee^	4.89 ± 0.02^Ea^	18.87 ± 0.90^Ed^	12.52 ± 0.05^Ec^	8.52 ± 0.03^Eb^
Total oxidation value—Totox					
0	5.93	5.73	5.86	5.81	5.72
7	141.42	7.29	7.59	7.31	7.01
14	196.23	7.58	19.81	19.43	16.37
21	86.92	15.30	55.91	32.12	29.36
28	101.28	17.83	195.81	124.32	95.82
Induction time (h)					
0	4.15 ± 0.04^Ba^	14.92 ± 0.04^Be^	8.13 ± 0.02^Bb^	12.00 ± 0.03^Bc^	12.96 ± 0.04^Bd^
28	1.53 ± 0.02^Aa^	4.99 ± 0.02^Ae^	1.91 ± 0.02^Ab^	2.05 ± 0.02^Ac^	2.87 ± 0.02^Ad^

The values are mean ± SD. Mean values relating to analysed property of lipid fractions extracted from sponge-fat cakes depending on the cakes formula marked by the different lower-case superscripts letters (a–e) within a row denote statistically significant differences (*p* < 0.05). Value relating to analysed property of lipid fractions extracted from sponge-fat cakes depending on the storage time of cakes marked by the different upper-case superscripts letters (A–E) within a column denote statistically significant differences (*p* < 0.05)

### Changes in PV and *p*-AV values during storage of the sponge-fat cakes

During fat extraction and processing and also storage of foods containing fat, fat oxidation and formation of primary and secondary oxidation products are possible. Primary lipid oxidation products such as hydroperoxides are unstable, sensitive to temperature, very labile and can undergo degradation to generate a complex mixture of secondary products such as aldehydes, ketones, alcohols, and esters responsible for deterioration of organoleptic properties of food rich in fat (Gharby et al. 2016). PV measurement is the most common method of determining the content of hydroperoxides. In turn, *p*-AV is used as an indicator of concentration of secondary lipid oxidation products. The PV values for lipid fractions extracted from the sponge-fat cakes directly after baking ranged from 0.88 to 0.99 meq O_2_/kg (Table 2). According to the Polish Standard (PN-A-86902:^1997^), these values for bakery fats should not exceed 3 meq O_2_/kg. Higher PVs were observed for all lipid fractions extracted from the sponge-fat cakes without and with the addition of antioxidants during storage at 63 °C. For the control sample (without antioxidants), PV increased dramatically after 7 days of storage, reaching 63.45 meq O_2_/kg and suggesting that lipids in that sample were significantly oxidized. After 7 consecutive days of the control sample storage, PV increased to reach its maximum value (89.55 meq O_2_/kg) and then started to decline during further storage. The content of primary lipid oxidation products in the control sample after 28 days of storage was around 18 times higher than the value found for lipids extracted immediately after baking of the sponge-fat cakes. The incorporation of BHA into the sponge-fat cakes showed a gradual increase of PVs for lipid fractions extracted in the entire storage period. The rate of hydroperoxides formation accelerated about twice after 7 and 14 days of the cakes’ incubation and nearly 7 times after 21 and 28 days of storage, respectively. The values of PV were lower than those for the control sample, that is, within the range from 1.63 (after 7 days of storage) to 6.47 meq O_2_/kg (after 28 days of storage). These results indicated that the addition of BHA to cakes may contribute to a better protection of lipids against the oxidation process and against unfavourable sensory changes during storage of food rich in fat.

At present, a consumer does not accept synthetic food additives, therefore the use of natural additives, such as green tea extracts may be an alternative. Tea polyphenols are a suitable mixture of natural antioxidants capable of scavenging oxygen radicals and of chelation of metal ions (Taghvaei and Jafari 2015). The green tea polyphenols also show stronger antioxidative activity compared to BHA and tocopherol (Koketsu and Satoh 1997). In our study, PV values for lipids extracted from the sponge-fat cakes enriched with green tea extracts after baking were lower than for the samples prepared from the cakes without additives. This may suggest that fat was protected by green tea extracts during preparation of the dough and baking. By the 21st day of storage at 63 °C of the sponge-fat cakes with 3 different concentrations of the green tea extract, significantly lower values of PV were observed for lipid fractions than for their counterparts extracted from the cakes without natural antioxidants. However, the PV values for lipids extracted from the cakes containing 1% green tea extract changed the least. Mildner-Szkudlarz et al. (2009) also confirmed protective effects of 1% green tea extracts and BHA in inhibiting hydroperoxides formation after addition to biscuits and their incubation during 20 days at 60 °C. However, their peroxide values were much lower compared to our investigations. The lower PV could be influenced by the type of fat used, the manner of biscuit storage (in extra foil bags), and the size of stored cookies (6 cm). Nonetheless, our samples enriched with green tea extracts which were stored at 63 °C for up to 28 days were characterized by a higher rate of peroxide formation than the control sample. These observations indicate that the tea extracts may also act as pro-oxidants. The PV was also higher in the olive oil with a green tea aqueous extract compared to the control sample, when samples were exposed to increased heat (Malheiro et al. 2012). In addition, the lower PV of the lipid fraction extracted from the sponge-fat cakes without the green tea extract addition, after 28 days of storage, may indicate that the large quantities of hydroperoxides formed after 14 days of cakes storage could be decomposed into secondary compounds.

The determination of the secondary lipid oxidation products was possible using *p*-AV. For all the studied samples which were incubated at 63 °C for 28 days, increasing *p*-anisidine values were observed (Table 2). The highest *p*-AV was detected in samples without antioxidants. It increased from 3.95 (after baking) to 66.38 during 28 days of storage. In contrast to the control sample, the content of secondary lipid oxidation products was lower for the samples treated with BHA and green tea extracts. Addition of BHA to the sponge-fat cakes caused a significant reduction of *p*-AV in the lipid fraction compared to the control sample starting from baking to the end of storage (from 3.95 to 4.89). The samples with the addition of green tea extracts also had significantly lower *p*-AV than the control sample. The best results were obtained in the samples containing 1% green tea extract, but they did not inhibit the formation of peroxide product decomposition during storage more effectively than the samples with BHA. This observation was in agreement with those reported by Mildner-Szkudlarz et al. (2009). In our investigation, however, the antioxidative effect of green tea extracts used in preparation of the sponge-fat cakes at concentrations of 0.02%, 0.2% and 1% was 3.5, 5 and 8 times higher compared to the control sample until the end of their storage.

PV in conjunction with *p*-AV are often used to calculate a degree of fat oxidation (Totox). In our study, the same tendency as for PV was observed for Totox but the obtained values were higher. The control sample’s Totox value was the highest and amounted to 196.3 after 14 days of storage, while the presence of BHA and green tea extracts protected lipids to a higher degree (7.58 and 16.37–19.81 of Totox values, respectively). The substantial decrease of PV for lipids extracted from the control sample after 28 days of storage at 63 °C and the lower increase of secondary lipid oxidation products at the same time caused a decrease in Totox value (*p* < 0.05). This may be an argument supporting the conclusion that the use of Totox in our research as the main parameter describing the overall degree of fat oxidation would be inappropriate. However, the determination of peroxide and *p*-anisidine values helps to assess the quality of fat and fat-containing food and to provide the initial information about oxidative degradation of fats.

### Thermal oxidative stability of lipid fraction

Lipid oxidation compromises the quality and limits the shelf life of various food products (Daglioglu et al. 2004). Methods such as Rancimat and DSC are widely applied in the determination of fat oxidative stability. Rancimat test allows to evaluate the induction time on the basis of the increase of water conductivity as a result of the oxidation process. The longer the induction time, the longer the durability of fat. The highest induction time for lipid fraction extracted from the sponge-fat cakes immediately after baking was observed for samples containing BHA and green tea extract at a concentration of 1% (Table 2). The addition of 1% green tea extract improved the stability of lipid fraction compared to the control sample from 4.15 to 12.96 h. Similar observations were also reported by Żbikowska et al. (2017). Sponge-fat cakes enrichment with lower concentrations of green tea extracts also prolonged the induction time of the lipid fraction when compared to the sample without any added antioxidants. The induction time values of these samples were 8.13 h (0.02%) and 12 h (0.2%), respectively. After 28 days of storage, the thermal stability of the studied samples measured by induction time decreased but the presence of antioxidants still displayed positive effects. Induction times of all the samples enriched with BHA and green tea extracts were higher than of those without additives. According to Gramza-Michlowska et al. (2007), green tea extract also protects lipids from oxidation for nearly three times and rosemary extract for two times longer than is the case in the control sample. The extension of induction time by addition of green tea extracts is related to their antioxidant efficacy corresponding to polyphenols content. Green tea catechins have the ability to scavenge free radicals or to chelate metal ions. They trap hydroxyl radicals and superoxide anions, suppress and then terminate the free radical chain reaction occurring during lipid peroxidation (Nikoo et al. 2018; Taghvaei and Jafari 2015).

In comparison to the Rancimat test, DSC can be applied in the evaluation of oxidative stability of samples containing volatile antioxidants and other lipid systems containing water (Gortzi et al. 2007). It is a method which does not require big samples and any chemicals or solvents and can provide reproducible data. The onset temperature (t_ON_) indicates the beginning of lipid oxidation and change in quality of fat (Tengku-Rozaina and Birch 2016). Generally, a sample with a higher t_ON_ is more stable than the one for which t_ON_ values obtained at the same heating rates are lower. The results listed in Table 3 showed that lipid fractions extracted from the sponge-fat cakes after baking had relatively high onset oxidation temperatures. However, addition of a 1% green tea extract produced higher t_ON_ values than in the control sample at the same heating rate (p < 0.05). For all the samples tested, the increase in heating rate from 4 to 15 °C min^−1^ led also to an increase in t_ON_ values. Similar observations were made for oxidation of camelina seed oils obtained by different extraction methods and for oxidation of fat extracted from cookies fortified with green, nettle and black currant seeds extracts (Belayneh et al. 2017; Zbikowska et al. 2018). After 7 and 14 days of cakes storage at 63 °C, a large decrease of t_ON_ for the lipid fractions without additives was observed. The samples enriched with BHA and green tea extracts were characterized by a greater stability because of higher t_ON_ values than for the control sample. These data are in agreement with those presented for PV values, which increased significantly for the control sample and slightly for samples enriched with antioxidants at the same time of storage. A decline of the onset temperature was observed as the peroxide value increased. It may indicate a good correlation between these parameters. Likewise, both induction times measured by Rancimat and the oxidation onset temperatures slightly decreased for the lipid fractions extracted from the cakes enriched with BHA and green tea extracts compared to the control sample after 28 days of storage. Among the green tea extracts used to fortify the cakes, 1% tea extracts showed the best protective effect in inhibiting hydroperoxides formation during 28 days of the samples storage. This observation was in agreement with kinetic parameters calculated from experimental DSC data (t_ON_), such as the Arrhenius activation energies, pre-exponential factors and, above all, induction times (τ) at 150 °C (Table 4). The Arrhenius activation energies of the samples studied after baking were with the range of 120.16-152.16 kJ mol^−1^, and after 28 days of storage they varied from 101.89 kJ mol^−1^ for the control sample to 125.81 kJ mol^−1^ for the sample enriched with 1% green tea extract. Kozłowska et al. (2014) obtained a lower activation energy (81.13 kJ mol^−1^) for the lipid fraction extracted from a control sample after baking. It may be associated with a different content of saturated (10% in Kozlowska et al.) and unsaturated (90%) fatty acids in the composition of fat used for preparation of the cakes. In our studies, fat used in the formulation of sponge-fat cakes contained 70% of unsaturated and 30% of saturated fatty acids. The presence of a lower number of double bonds in the composition of fatty acids was conducive to oxidation of fats at higher temperatures. Based on the induction time (τ values) calculated for lipid fractions extracted from all kind of cakes after baking, we can rank their stability in the following sequence: green tea extract 1% > BHA > green tea extract 0.2% > green tea extract 0.02% > control. After 21 days of storage, resistance of lipid fraction to thermo-oxidative decomposition increased in the following sequence: green tea extract 0.02% < green tea extract 0.2% < control < green tea extract 1% < BHA.

**Table 3:** Extrapolated DSC thermooxidation onset temperatures (t_ON_/°C) measured at different heating rates (*β*) for studied samples

Heating rate, *β* (°C min^−1^)	Control	BHA 0.02%	Green tea extract		
			0.02%	0.2%	1%
After baking					
4.0	172.21 ± 0.88^Aa^	175.89 ± 1.11^Ab^	173.63 ± 0.54^Ab^	174.80 ± 0.44^Ac^	177.10 ± 0.88^Ae^
7.5	181.18 ± 0.96^Bb^	184.01 ± 0.99^Be^	180.33 ± 0.52B^a^	182.30 ± 0.57^Bc^	183.26 ± 0.93^Bd^
10.0	185.44 ± 1.18^Cc^	186.58 ± 1.45^BCd^	183.68 ± 0.85^Ca^	184.47 ± 0.87^Bb^	187.09 ± 0.94^Ce^
12.5	187.42 ± 1.22^Cb^	188.91 ± 0.94^CDc^	186.39 ± 0.66^CDa^	187.43 ± 1.03^Cb^	189.44 ± 1.13^CDd^
15.0	190.14 ± 1.45^Ca^	191.17 ± 1.22 ^Da^	189.12 ± 0.96 ^Da^	188.93 ± 1.27^Ca^	191.31 ± 1.09 ^Da^
After 7 days of storage					
4.0	131.11 ± 0.72^Aa^	148.15 ± 0.56^Ab^	164.64 ± 0.75^Ac^	173.10 ± 0.65^Ad^	173.46 ± 0.63^Ad^
7.5	138.05 ± 0.84^Ba^	155.99 ± 0.57^Bb^	171.89 ± 0.66^Bc^	181.46 ± 0.79^Be^	180.94 ± 0.78^Bd^
10.0	143.03 ± 1.05^Ca^	159.99 ± 0.73^Cb^	174.97 ± 0.78^Cc^	185.40 ± 0.86^Ce^	183.93 ± 0.93^Cd^
12.5	146.45 ± 0.98^Ca^	163.98 ± 0.88^Db^	178.32 ± 0.83^Dc^	190.30 ± 0.95^De^	186.48 ± 1.11^Dd^
15.0	149.93 ± 1.12^Ca^	167.12 ± 1.28^Eb^	181.44 ± 1.28^Ec^	192.12 ± 1.17^Ee^	188.59 ± 1.06^Ed^
After 14 days of storage					
4.0	135.21 ± 0.36^Aa^	169.33 ± 0.46^Ae^	158.44 ± 0.65^Ab^	164.82 ± 0.51^Ac^	168.79 ± 0.62^Ad^
7.5	144.50 ± 0.47^Ba^	180.43 ± 0.67^Bb^	167.89 ± 0.69^Bb^	175.38 ± 0.73^Bb^	176.68 ± 0.79^Bb^
10.0	145.97 ± 0.83^Ca^	184.26 ± 0.83^Cd^	172.41 ± 0.79^Cb^	179.95 ± 0.89^Cc^	180.03 ± 0.93^Cc^
12.5	149.03 ± 0.98 ^Da^	187.31 ± 0.88^Dd^	177.19 ± 0.93^Db^	183.95 ± 1.16^Dc^	183.64 ± 1.15^Dc^
15.0	151.32 ± 1.08^Ea^	190.52 ± 1.18^Ee^	181.03 ± 1.11^Eb^	186.02 ± 1.18^Ec^	187.04 ± 1.37^Ed^
After 21 days of storage					
4.0	161.64 ± 0.56^Ac^	174.34 ± 0.57^Ae^	153.80 ± 0.52^Aa^	158.19 ± 0.48^Ab^	163.14 ± 0.57^Ad^
7.5	169.09 ± 0.68^Bc^	183.51 ± 0.77^Be^	162.52 ± 0.63^Ba^	164.56 ± 0.54^Bb^	171.63 ± 0.69^Bd^
10.0	174.35 ± 0.89^Cc^	187.14 ± 0.96^Cd^	165.44 ± 0.73^Ca^	168.21 ± 0.69^Cb^	174.37 ± 0.87^Cc^
12.5	176.94 ± 1.22^Dc^	190.98 ± 1.07^De^	168.14 ± 0.94 ^Da^	170.27 ± 0.89^Db^	177.97 ± 1.05^Dd^
15.0	179.96 ± 1.48^Ec^	193.39 ± 1.21^Ee^	171.15 ± 0.95^Ea^	173.69 ± 0.91^Eb^	181.15 ± 1.15^Ed^
After 28 days of storage					
4.0	161.41 ± 0.46^Ae^	161.05 ± 0.74^Ad^	144.55 ± 0.37^Aa^	148.75 ± 0.43^Ab^	150.69 ± 0.52^Ac^
7.5	169.53 ± 0.58^Be^	168.35 ± 0.95^Bd^	153.06 ± 0.48^Ba^	155.39 ± 0.54^Bb^	157.65 ± 0.63^Bc^
10.0	174.67 ± 0.79^Ce^	172.48 ± 1.06^Cd^	155.98 ± 0.62^Ca^	158.83 ± 0.59^Cb^	161.30 ± 0.73^Cc^
12.5	178.36 ± 1.12^De^	174.82 ± 1.17^Dd^	159.67 ± 0.86 ^Da^	161.67 ± 0.85^Db^	163.85 ± 0.94^Dc^
15.0	181.51 ± 1.38^Ee^	178.51 ± 1.31^Ed^	162.07 ± 0.95^Ea^	164.75 ± 0.93^Eb^	166.13 ± 0.95^Ec^

The values are mean ± SD. Mean values relating to analysed property of lipid fractions extracted from sponge-fat cakes depending on the cakes formula marked by the different lower-case superscripts letters (a–e) within a row denote statistically significant differences (*p* < 0.05). Value relating to analysed property of lipid fractions extracted from sponge-fat cakes depending on the storage time of cakes marked by the different upper-case superscripts letters (A–E) within a column denote statistically significant differences (*p* < 0.05)

**Table 4:** Kinetic parameters characterizing the thermooxidation of samples studied

Parameters	Control	BHA 0.02%	Green tea extract		
			0.02%	0.2%	1%
After baking					
a and b	6.599 and 15.41	7.903 and 18.19	7.727 and 17.91	8.357 and 19.25	8.314 and 19.07
r^2^	0.996	0.995	0.998	0.995	0.998
E_a_ (kJ mol^−1^)	120.16	143.90	140.69	152.16	151.39
log Z	13.57	16.27	15.99	17.30	17.13
τ at 150 °C (min)	18.35	31.25	23.81	30.49	36.23
After 7 days of storage					
a and b	5.179 and 13.44	5.620 and 13.96	6.901 and 16.38	6.110 and 14.30	7.848 and 18.17
r^2^	0.990	0.995	0.995	0.994	0.999
E_a_ (kJ mol^−1^)	94.31	102.33	125.65	111.26	142.89
log Z	11.70	12.18	14.51	12.49	16.25
τ at 150 °C (min)	0.87	2.83	10.02	17.57	24.39
After 14 days of storage					
a and b	6.229 and 15.84	5.604 and 13.26	5.005 and 12.21	5.349 and 12.81	6.494 and 15.31
r^2^	0.983	0.995	0.995	0.998	0.995
E_a_ (kJ mol^−1^)	113.42	102.03	91.14	97.40	118.25
log Z	14.02	11.48	10.49	11.06	13.47
τ at 150 °C (min)	0.96	12.99	5.78	9.17	13.42
After 21 days of storage					
a and b	6.113 and 14.67	6.275 and 14.63	6.396 and 15.58	7.272 and 17.48	6.438 and 15.36
r^2^	0.995	0.999	0.996	0.993	0.995
E_a_ (kJ mol^−1^)	111.30	114.26	116.45	132.41	117.22
log Z	12.86	12.80	13.74	15.59	13.53
τ at 150 °C (min)	7.57	20.20	4.31	5.71	8.69
After 28 days of storage					
a and b	5.596 and 13.49	6.557 and 15.71	5.981 and 14.92	6.710 and 16.52	6.910 and 16.91
r^2^	0.996	0.995	0.998	0.995	0.999
E_a_ (kJ mol^−1^)	101.89	119.38	108.90	122.17	125.81
log Z	11.72	13.87	13.12	14.67	15.04
τ at 150 °C (min)	7.19	7.35	2.11	2.57	3.10

Parameters *a*, *b* and *r*^*2*^ were obtained from plotting log *β* versus *T*_ON_^−1^ for all samples

E_a_ activation energy; log Z logarithm of pre-exponential factors (*Z*, min^−1^); *τ* induction time

Sometimes volatile components included in natural antioxidants can be lost during DSC analysis because of the high temperature at which antioxidants may be less stable and their effectiveness in protection of lipids may also be seriously undermined. Moreover, antioxidants from natural sources such as green tea extracts contain a mixture of several compounds with antioxidant function. Such extracts added to lipid-containing food may interact with each other and with antioxidants present in the food (Yin et al. 2012). A significant synergistic effects and sometimes also antioxidant antagonisms are possible. The strong antioxidant effect of green tea polyphenols was observed on lard and fish oil that are animal oils, soybean oil that is a vegetable oil, and on fried noodles (Koketsu and Satoh 1997). A green tea extract was also used to protect a Turkish dry-fermented sausage against oxidation at the time of ripening and it proved more effective than BHT (Taghvaei and Jafari 2015). Addition of a green tea powder to cookies resulted in a significant improvement of stability, viscoelastic and functional properties of wheat dough (Ahmad et al. 2015).

## Conclusion

Use of 1% green tea extract and BHA in sponge-fat cakes resulted in an improvement of the oxidative stability of lipid fractions extracted from these cakes. After baking, slight changes in PV, *p*-AV and AV values for all the tested samples were observed. However, the values of these parameters significantly increased in the sample without any additives compared to the samples enriched with 1% green tea extract and BHA during 21 days of storage. Lipid fraction extracted from the sponge-fat cakes containing green tea extract at the highest concentration and BHA were also characterized by higher t_ON_ values than the control sample obtained from dynamic DSC. These data prove that natural additives are very effective inhibitors of lipid oxidation and can be recommended for food products applications, especially those containing large amounts of fat.
